# Supplementary figures and images for: Human striatal organoids derived from pluripotent stem cells recapitulate striatal development and compartments
Source: PLoS Biol. 2022 Nov 17;20(11):e3001868. doi: 10.1371/journal.pbio.3001868 (PMC9714809; doi:10.1371/journal.pbio.3001868)

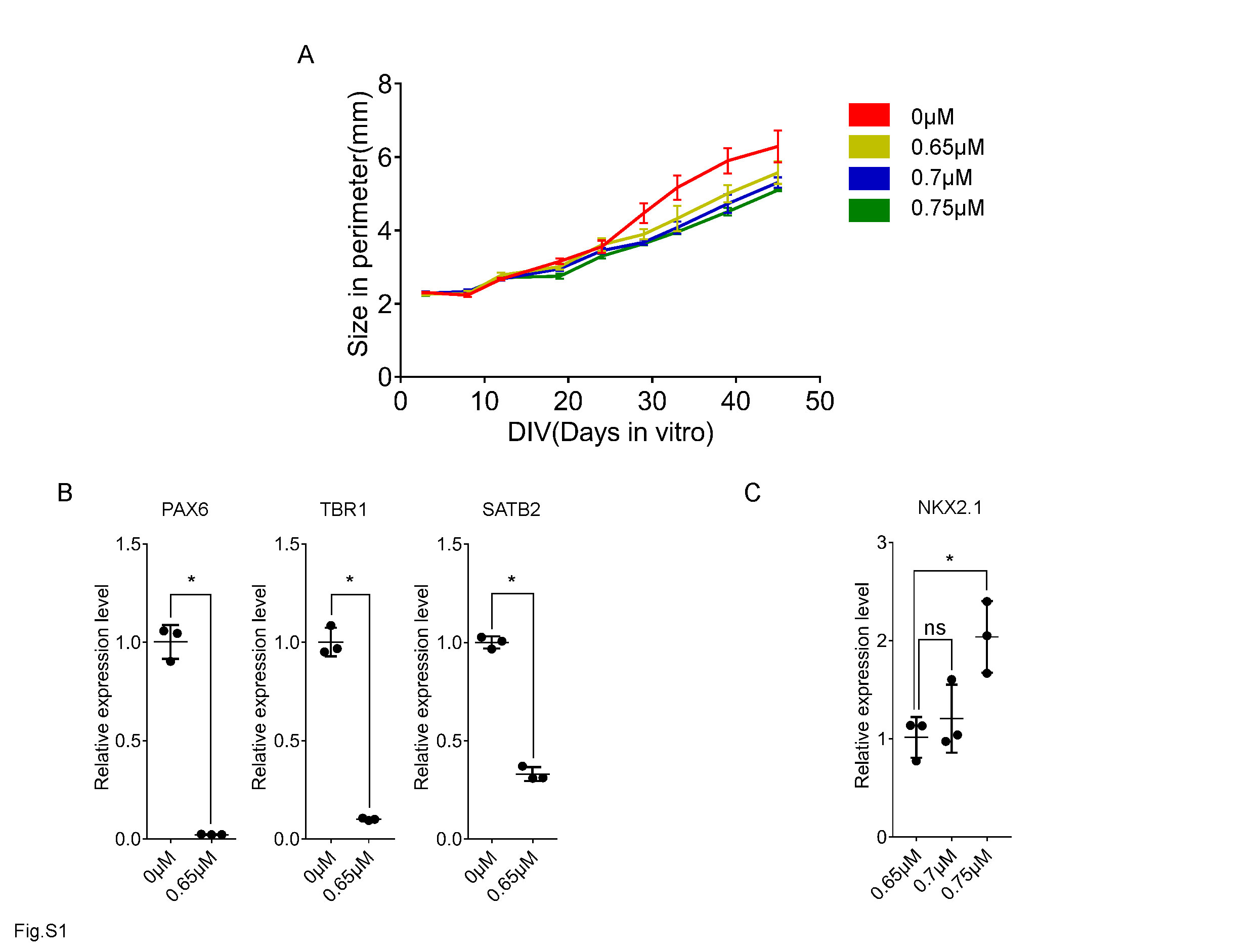

Supplement: S1 Fig — (A) Measuring the perimeters of organoid from Day 0 to Day 44 i. Data, mean ± SD; organoids, n = 4. (B) Evaluating PAX6, TBR1, and SATB2 transcription levels by qPCR. Data, mean ± SD; Student t test; *, P < 0.05. (C) Evaluating NKX2.1 transcription levels by qPCR. Values are plotted as the expression level (2-ΔCt) relative to the 0 μm group. Each data point corresponds to a pooled batch of 6 organoids. Data, mean ± SD. One-way ANOVA. *, P < 0.05, ns, no significant difference. The raw data underlying this figure can be found in the S2 Data. (TIF) [file pbio.3001868.s001.tif]

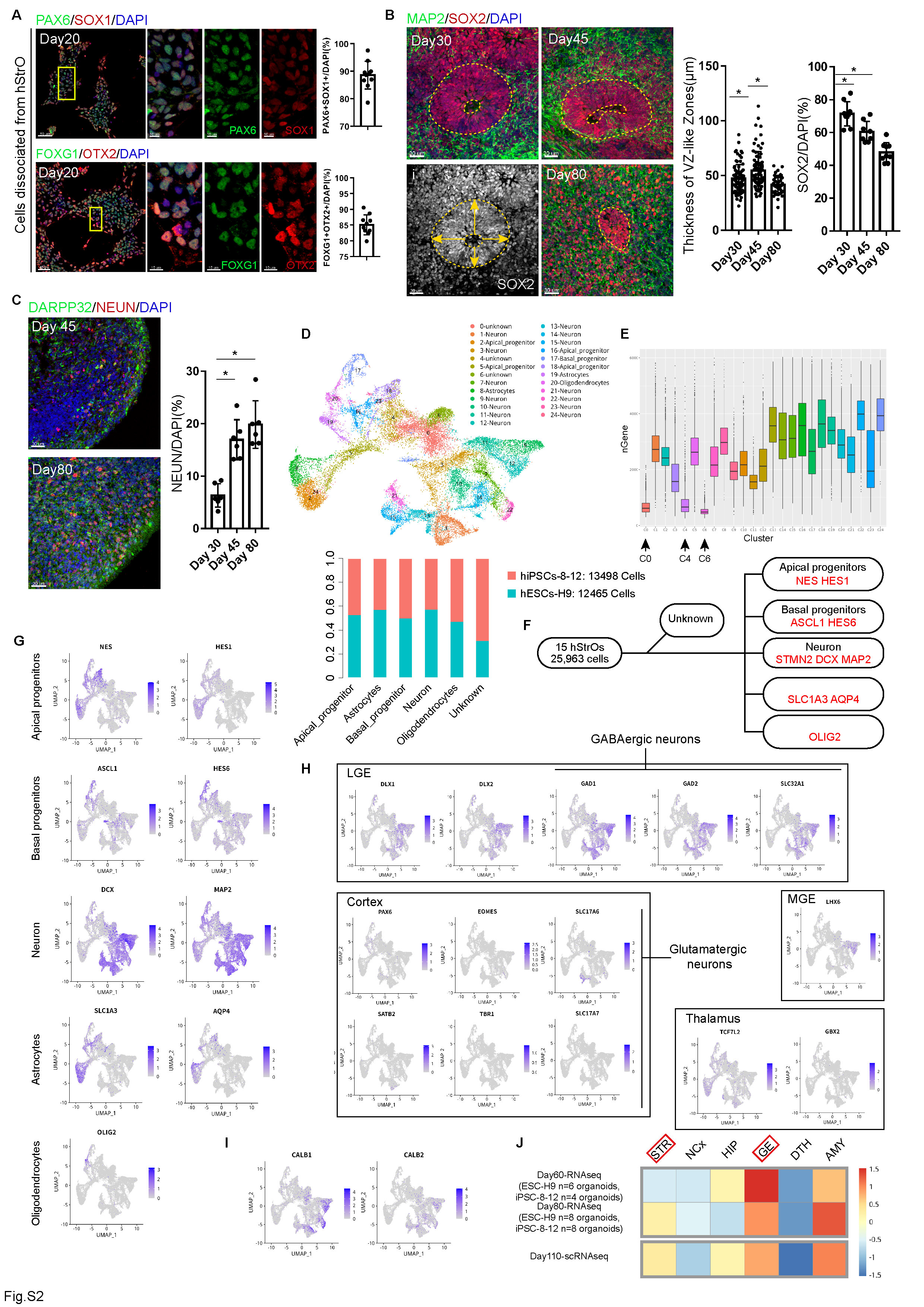

Supplement: S2 Fig — (A) The quantification of immunostaining with PAX6/SOX1 and OTX2/FOXG1 antibodies in the dissociated neurons from organoids revealed the telencephalic and the forebrain fates’ induction in hStrOs (Day 20). A total of 10 organoids were used in dissociated cultured in each group. Data, mean ± SD. Scale bar, upper 50 μm; lower 40 μm. (B) Immunostaining with SOX2 and MAP2 antibodies revealed multiple VZ-like areas in hStrOs in cultured. The mean thickness of each VZ-like area (indicated with the arrows in the schematic diagram) was used for quantification (organoids, n = 8). Quantification of SOX2+ cells on Day 30, Day 45, and Day 80 (n = 6 organoids). Data, mean ± SD. One-way ANOVA, *, P < 0.05. Scale bar, 30 μm. (C) Immunostaining with DARPP32 and NEUN antibodies in hStrOs revealed the mature striatal MSNs in hStrOs on Day 60 and Day 80. Quantification of NEUN+ cells at Day 30, Day 45, and Day 80 (n = 6 organoids). Data, mean ± SD. One-way ANOVA, *, P < 0.05. Scale bar, 30 μm. (D) UMAP visualization of the resolved scRNA-seq data of hStrOs. Histogram showing the percentage of cells in each cell type belonging to 2 cell lines in hStrOs (ESC-H9: green; iPSC-8-12: red). (E) Gene reads in each cluster. (F) Schematic representation of cluster annotation. (G–I) UMAP visualization of expression of selected genes in the hStrOs scRNA-seq data at Day 110 of in vitro differentiation (n = 26,534 cells from hESC-H9 and hiPSCs-8-12). (J) Bulk RNA-seq and scRNA-seq from hStrOs (Day 60, Day 80, and Day 110) mapped to the Brain Span human brain dataset (PCW8, 9, 12, 13, and 16) by using VoxHunt. The raw data underlying this figure can be found in the S2 Data. (TIF) [file pbio.3001868.s002.tif]

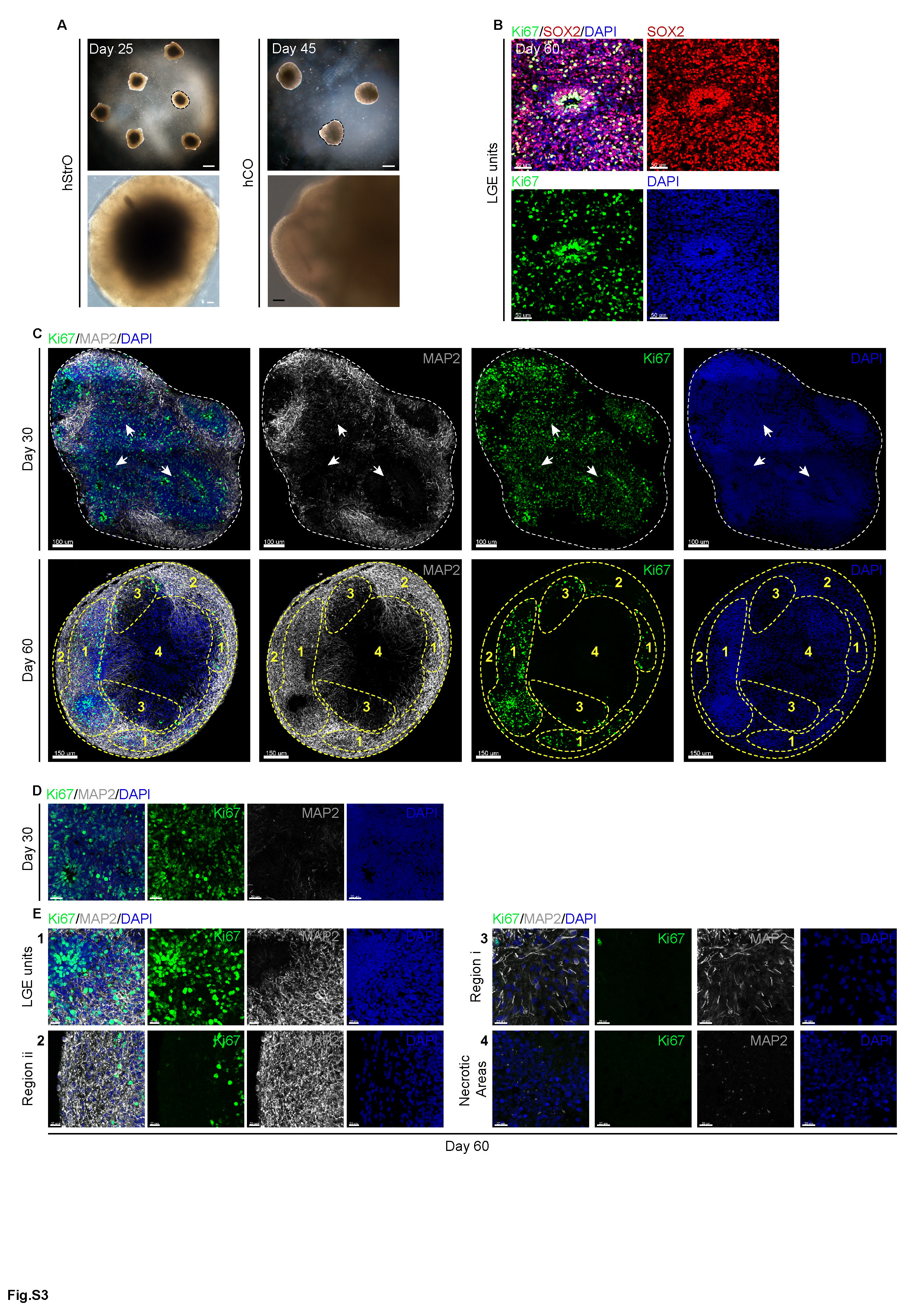

Supplement: S3 Fig — (A) The representative images of the hStrOs were collected on Day 25, and hCOs were collected on Day 45. Scale bar, 1 mm; insert, 100 μm. (B) Immunostainings for Ki67 and SOX2 antibodies revealed the expression of SOX2 and Ki67 in LGE units. Scale bar, 50 μm. (C–E) Immunostainings for Ki67 and MAP2 antibodies revealed progressive regionalization in Day 30 and 60 hStrOs. Arrows in C showed LGE units on Day 30 hStrO. Dashed lines marked the supposed region: (1) LGE units; (2) Region ii; (3) Region i; (4) Necrotic areas. Scale bar, C, above, 100 μm, down 150 μm; D, 30 μm; E, 20 μm. (TIF) [file pbio.3001868.s003.tif]

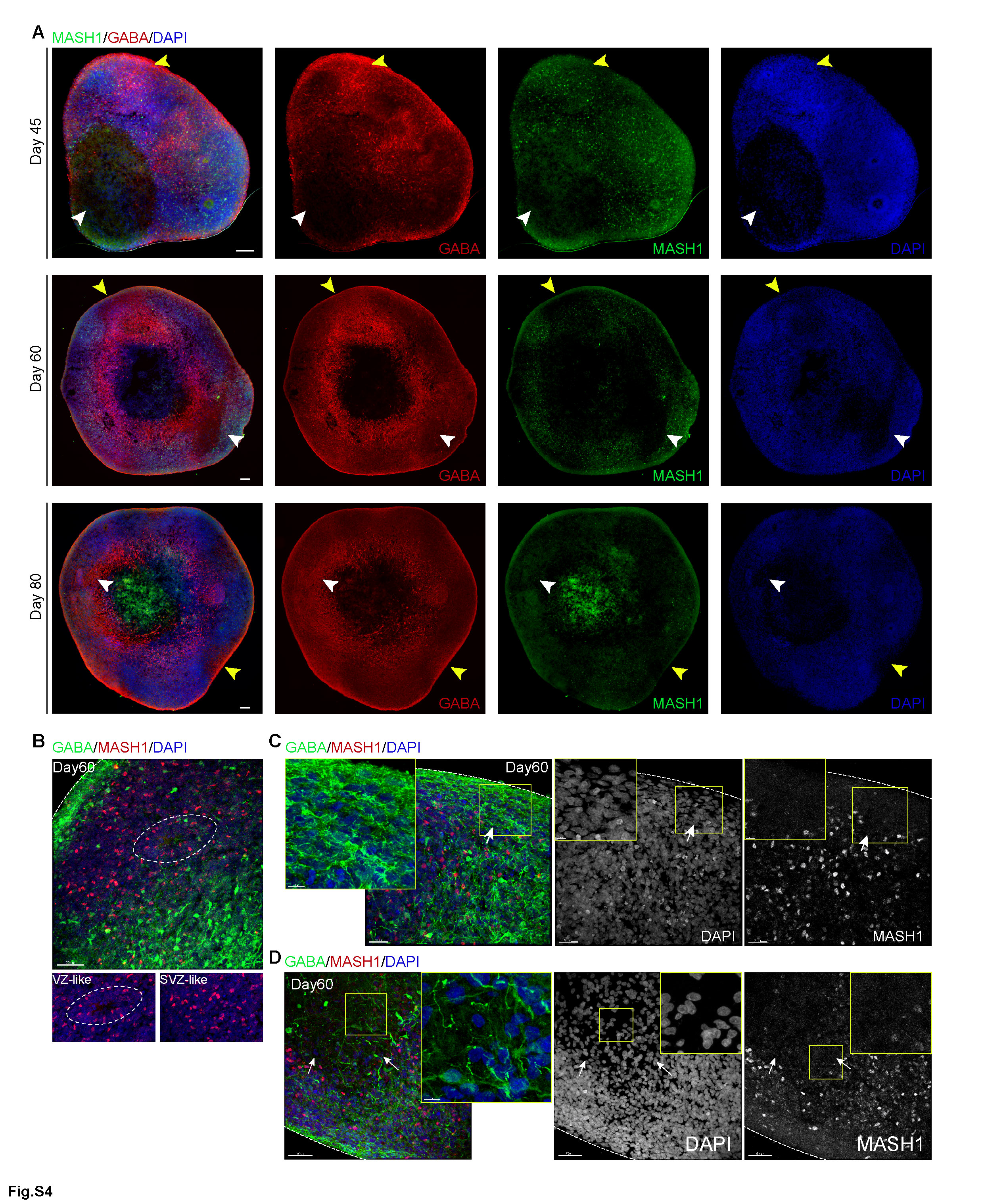

Supplement: S4 Fig — (A) Immunostainings for MASH1 and GABA antibodies revealed the distribution patterns of LGE progenitor cells on Day 45, Day 60, and Day 80 hStrOs. Arrows showed Region i (white) and Region ii (yellow). Scale bar, 100 μm. (B–D) Immunostainings for MASH1 and GABA antibodies revealed the distribution patterns of LGE progenitor cells in hStrOs. The LGE progenitor cells in hStrOs spatially separated from the mature neurons. Arrows showed the region that was absent from MASH1+ progenitor cells. Scale bar, B, 50 μm; C, 30 μm; D, 50 μm. (TIF) [file pbio.3001868.s004.tif]

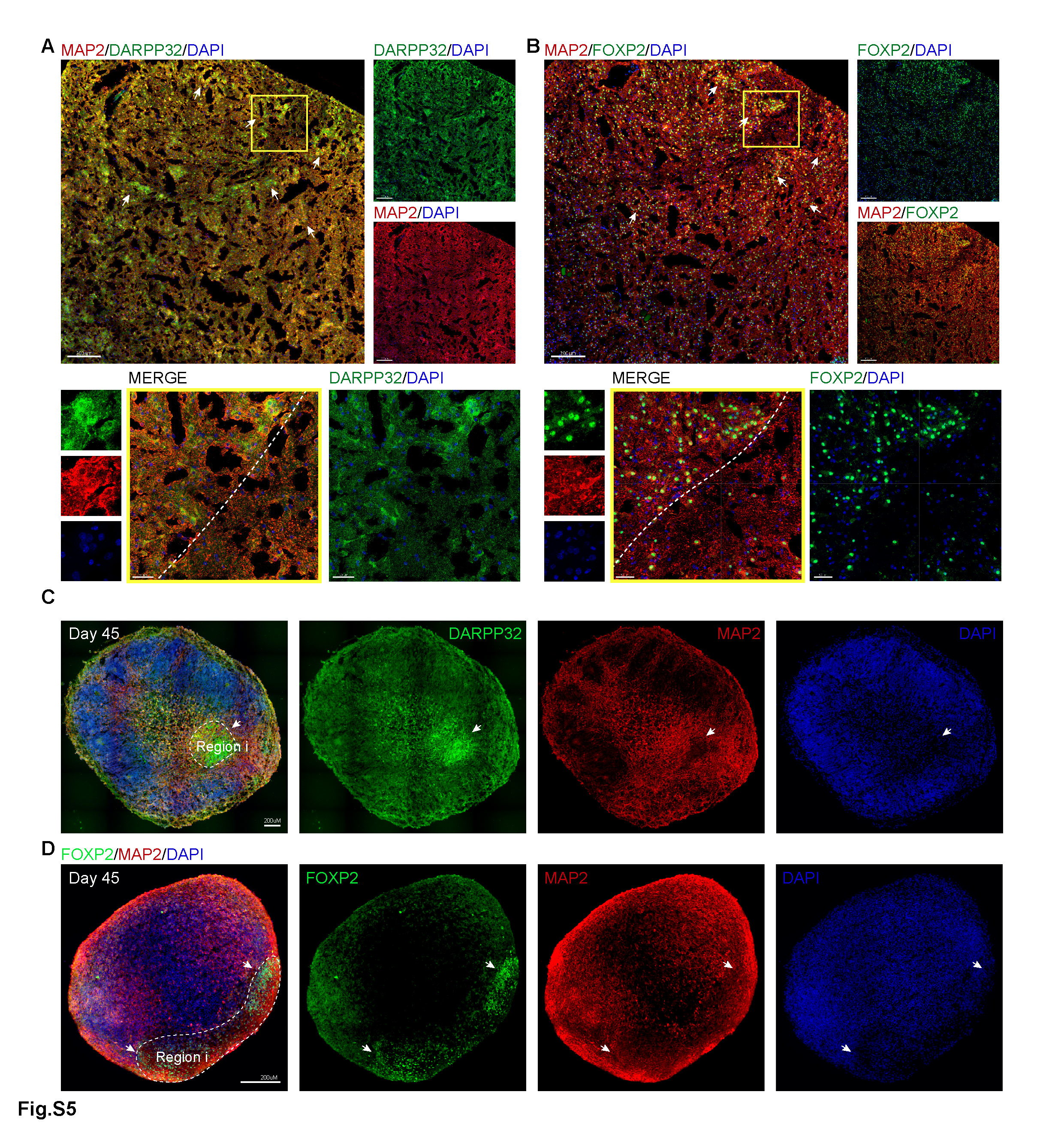

Supplement: S5 Fig — (A, B) Immunostaining with FOXP2, DARPP32, and MAP2 antibodies revealed the compartmentalization in the 22 W fetal striatum. Arrows showed mosaics embedded within the striatum formed by DARPP32+ or FOXP2+ patches. Scale bar, 300 μm; insert, 50 μm. (C, D) Immunostaining with FOXP2, DARPP32, and MAP2 antibodies revealed high expression of DARPP32 and FOXP2 in Region i of Day 45 hStrO. Arrows and dash line marked Region i. Scale bar, 200 μm. (TIF) [file pbio.3001868.s005.tif]

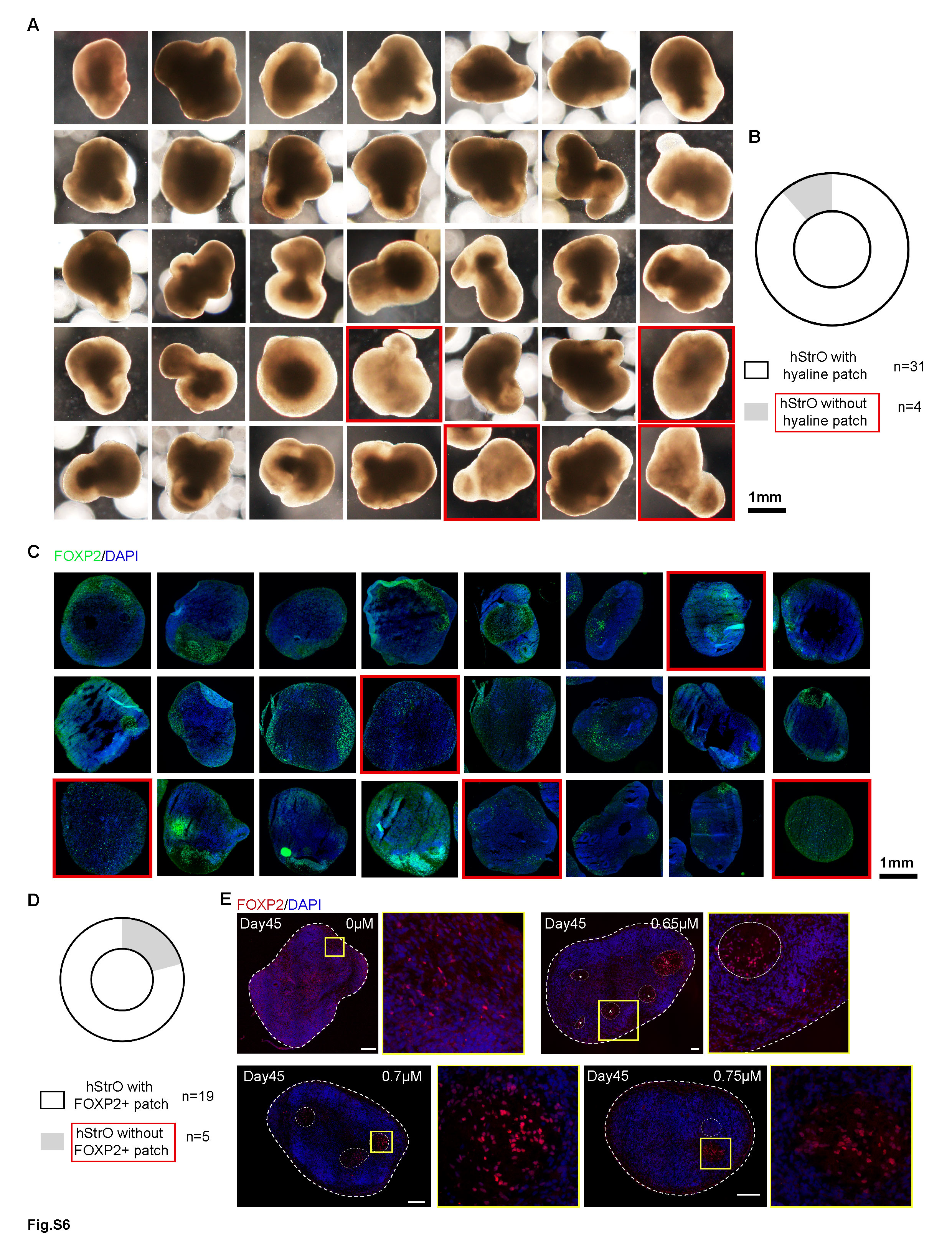

Supplement: S6 Fig — (A, B) Bright-field images showed that 89% of organoids (31/35) have a significant hyaline patch in a random batch of hStrO. Scale bar, 1mm. (C, D) Immunostaining with FOXP2 revealed that 79% of organoids (19/24) showed a transparent FOXP2 enriched Region i in random batches of hStrOs on Day 45. Scale bar, 1 mm. (E) Immunostaining with FOXP2 and MAP2 antibodies revealed the matrix striosome-like zones of organoids (Pur: 0.65, 0.7, and 0.75 μm), which is not observed in the organoid culture without Pur. Scale bar, 100 μm. (TIF) [file pbio.3001868.s006.tif]

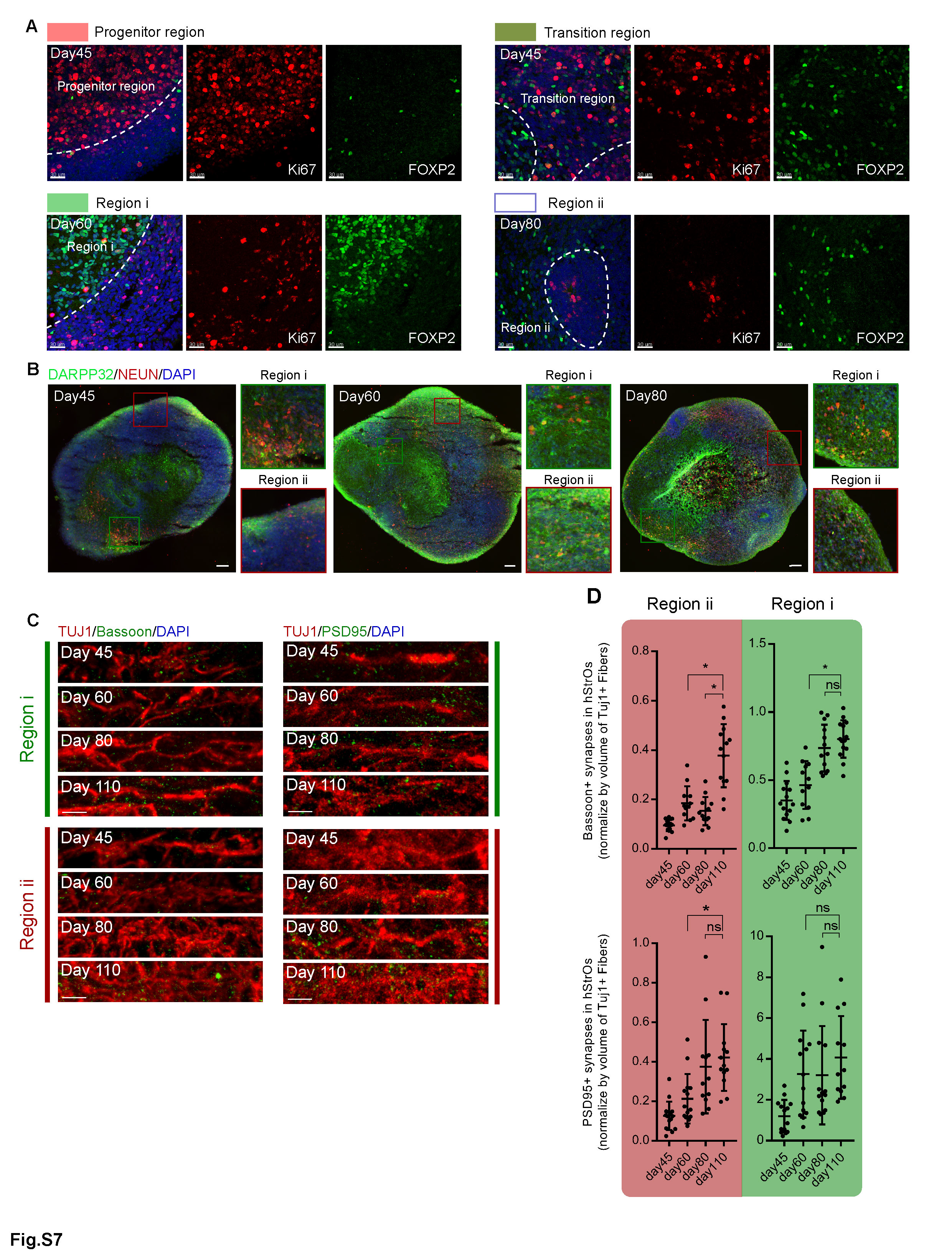

Supplement: S7 Fig — (A) Representative images showing 4 regions divided by Ki67 and FOXP2 expression in hStrOs. Scale bar, 30 μm. (B) Immunostaining of NeuN and DARPP32 antibodies revealed NEUN+DARPP32+ MSN in Mature regions i and ii on Day 45 to Day 80. Scale bar, 100 μm. (C) Immunostainings for Bassoon, PSD95, and Tuj1 antibodies reveal the existence of pre- and postsynaptic proteins in hStrOs after 45, 60, 80, and 110 days of culture. Scale bar, 5 μm. (D) Quantification of the number of Bassoon+ presynaptic and PSD95+ postsynaptic proteins in Mature region i and ii of hStrOs. Value on Y-axis = the counts of bassoon+ or PSD95+ puncta/volumes of Tuj1+ fibers rendered in IMARIS (n = more than 10 clear Tuj1+ fibers rendered in IMARIS). One-way ANOVA, *, P < 0.05; ns, nonsignificant. The raw data underlying this figure can be found in the S2 Data. (TIF) [file pbio.3001868.s007.tif]

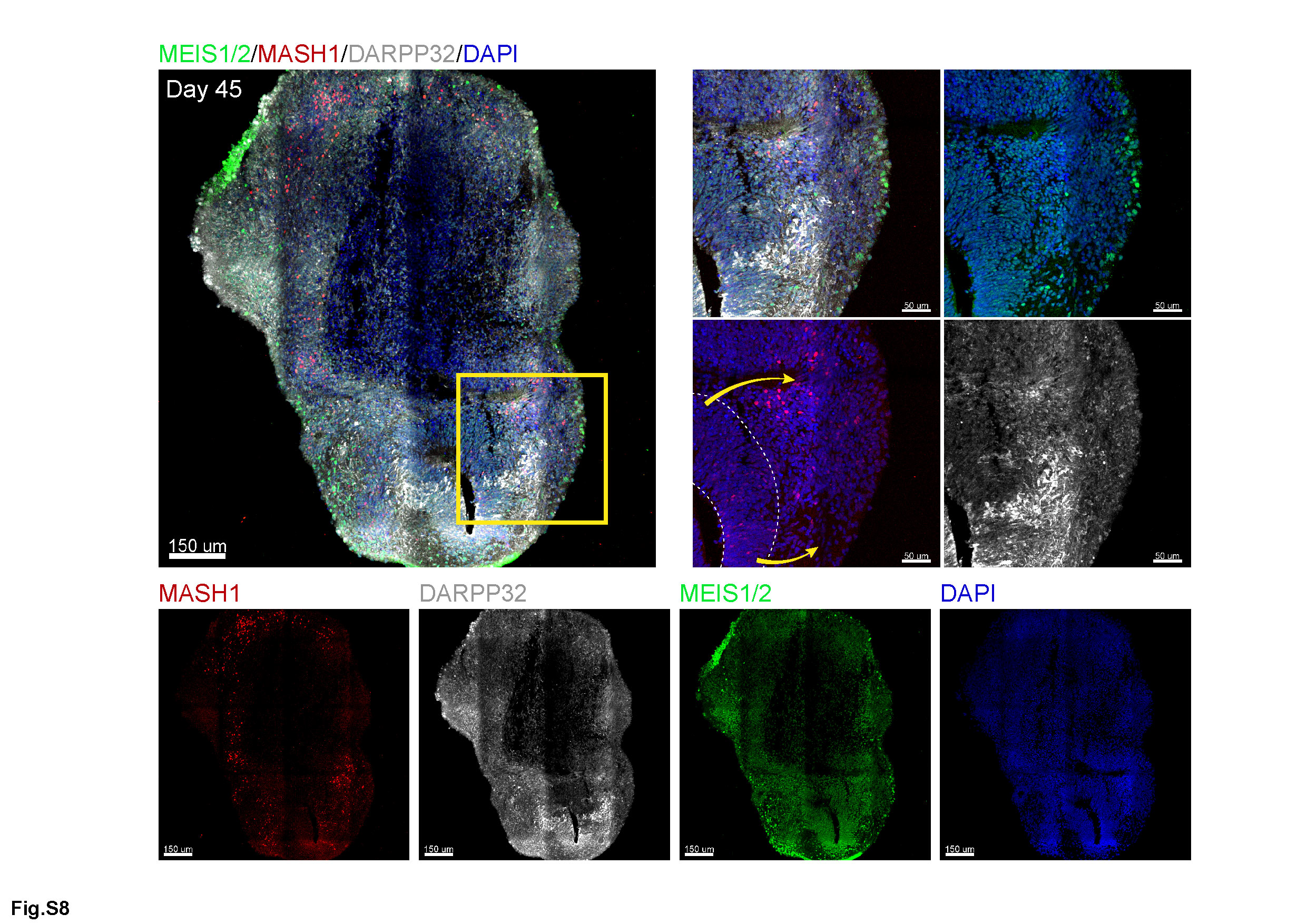

Supplement: S8 Fig — Immunostainings for MEIS1/2, MASH1, and DARPP32 reveal typical neural tubes contributing to Region i and LGE units in Day 45 hStrO. Arrows showed the possible migrating routes of progenitors from the individual rosette to participate in the hStrO regionalization. Scale bar, 150 μm; insert, 50 μm. (TIF) [file pbio.3001868.s008.tif]

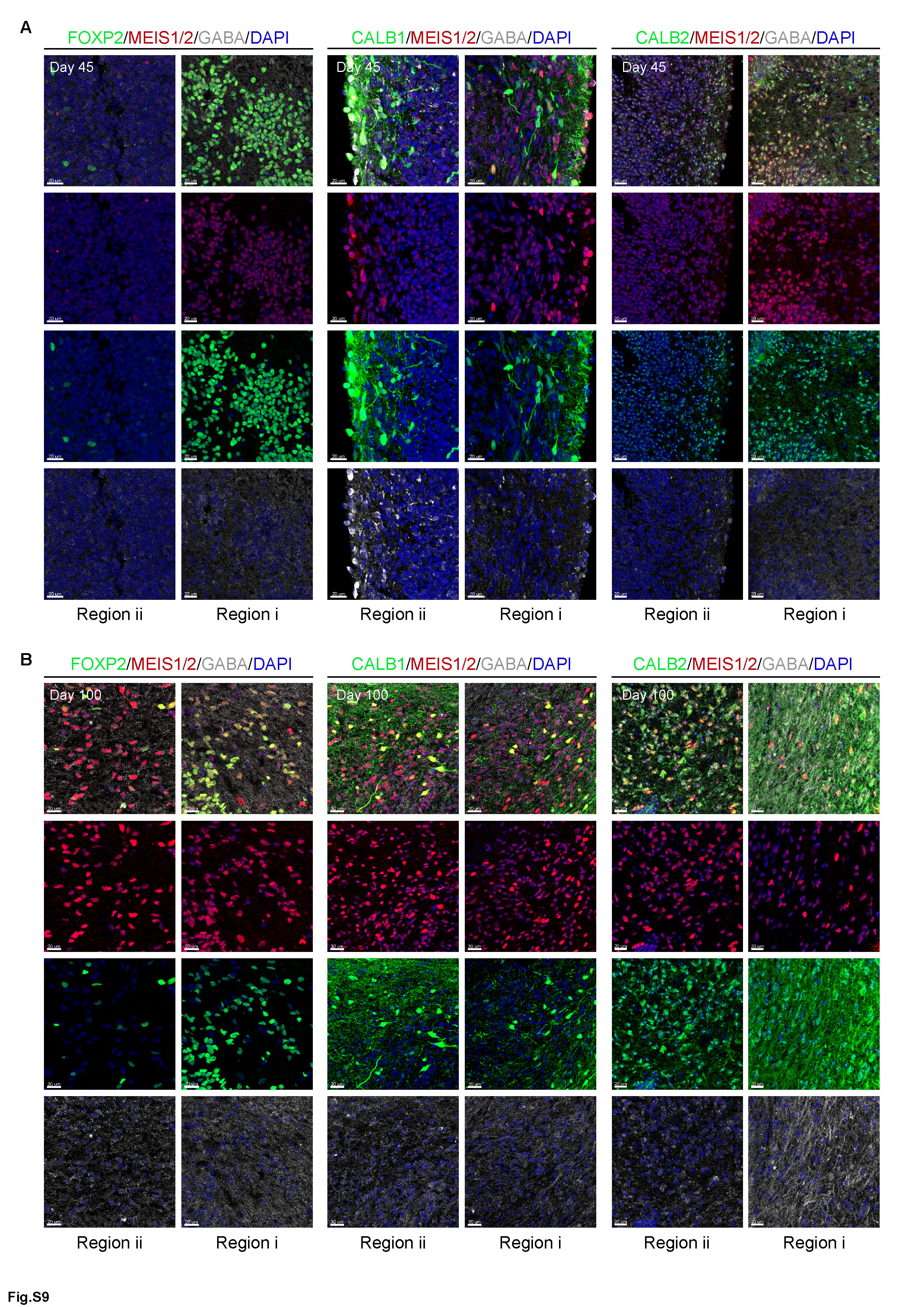

Supplement: S9 Fig — (A, B) Immunostainings for CALB1, CALB2, and FOXP2 antibodies on Day 45 and Day 100 hStrOs. Images showed typical CALB1/CALB2 expression in Region i/ii. Co-expressed of GABA and MEIS1/2 identified the striatal fates. Scale bar, A, 20 μm; B, 30 μm. (TIF) [file pbio.3001868.s009.tif]

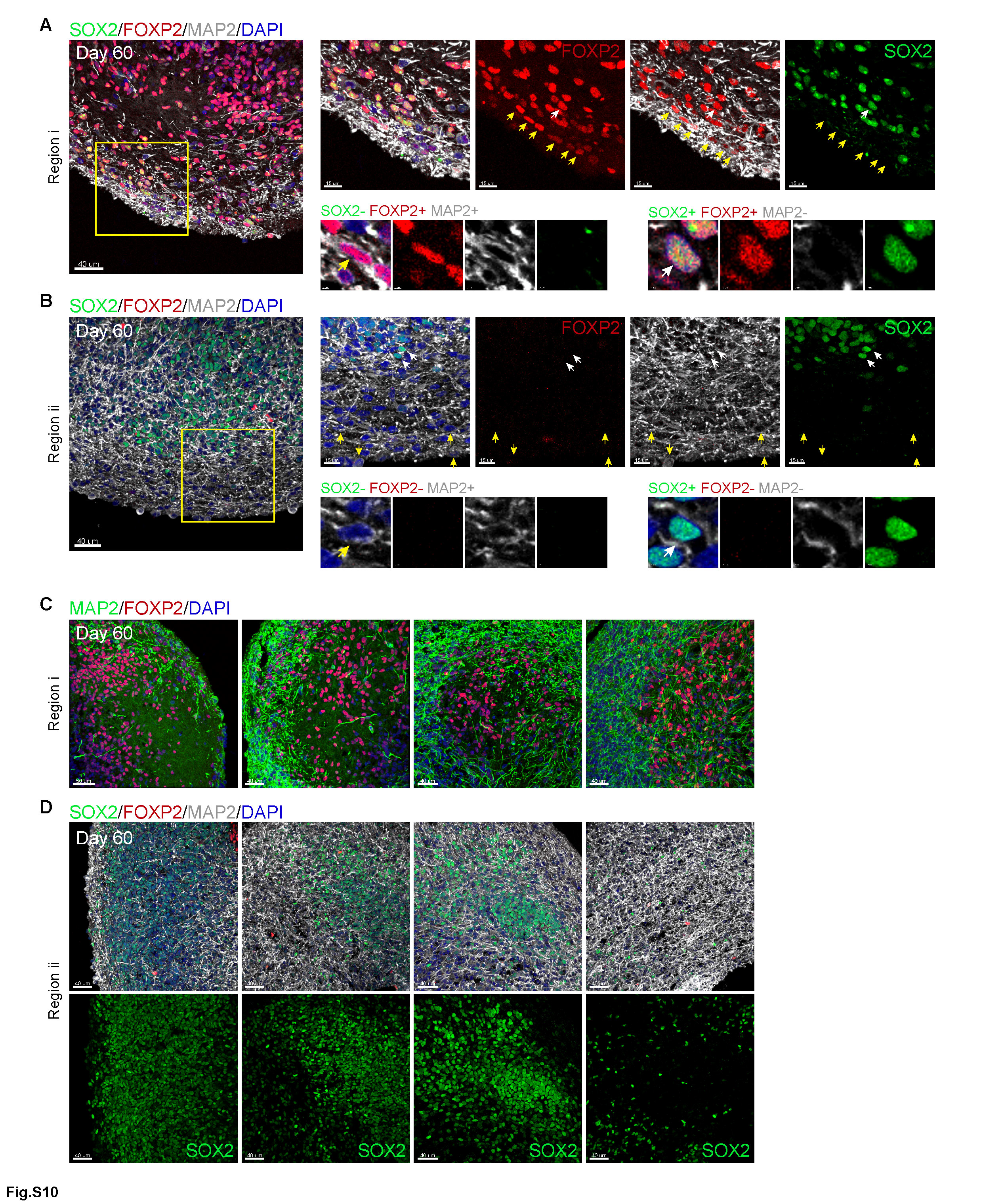

Supplement: S10 Fig — (A, B) Immunostainings for MAP2, SOX2, and FOXP2 antibodies revealed the mature neurons’ distribution in Region i and Region ii of Day 60 hStrOs. White arrows showed SOX2+ cells, and yellow arrows showed SOX2-MAP2+ mature neurons. The left panels are the magnified region from the boxed area, shown as 2 or spliced channels. Scale bar, 40 μm; insert, 15 μm, individual cell, 2 μm. (C, D) Immunostainings for MAP2, SOX2, and FOXP2 antibodies revealed mature neuronal distribution in different slices of the same organoids. From left to right, Region i/ii showed an increase in mature neurons. Scale bar, C, 50 μm; D, 40 μm. (TIF) [file pbio.3001868.s010.tif]

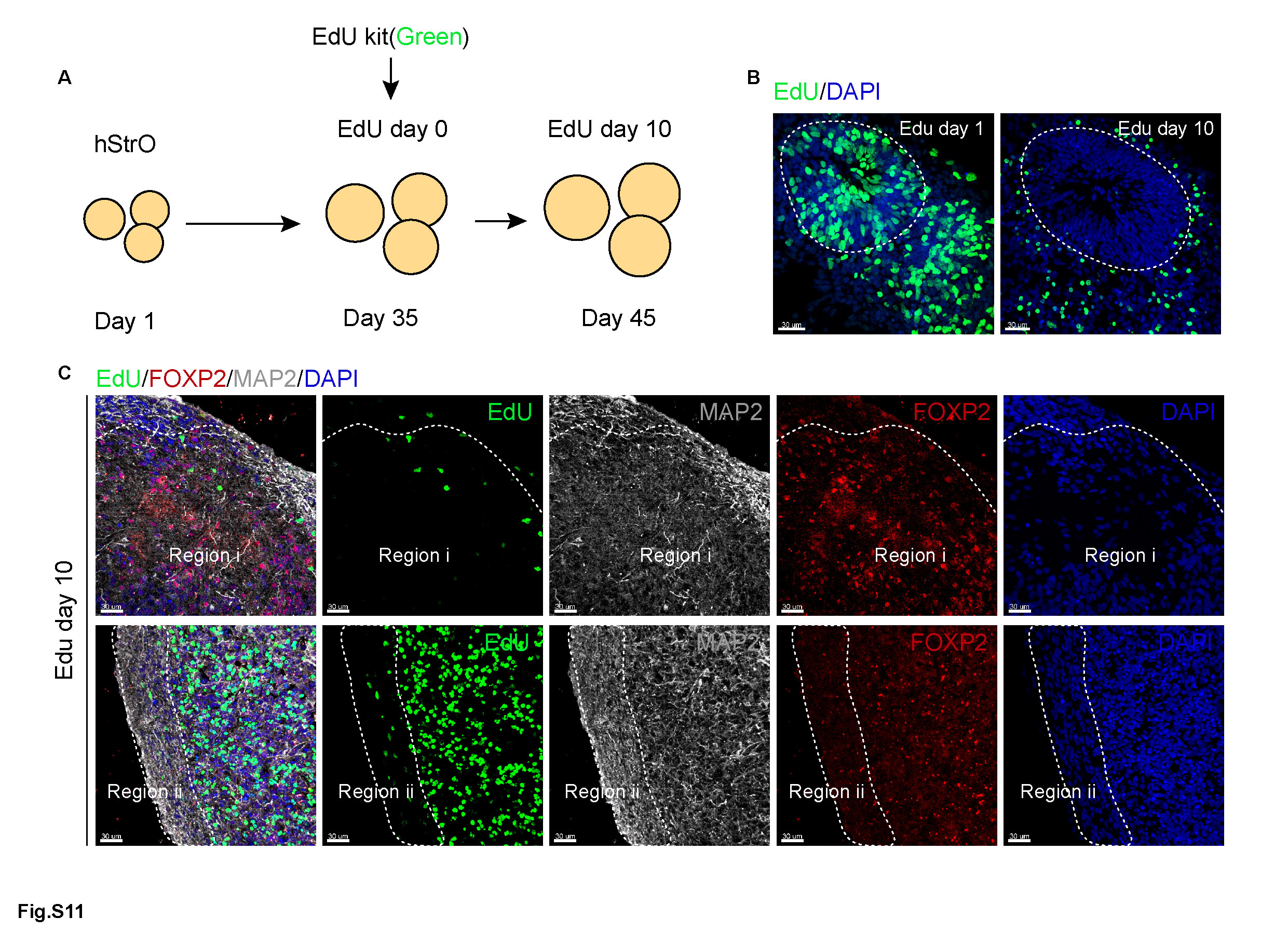

Supplement: S11 Fig — (A) Schematic of EdU tracing in hStrO. (B) EdU tracing showed EdU+ cells were predominantly identified in the rosette while excluded from the rosette after 10 days. Scale bar, 30 μm. (C) Immunostainings for MAP2 and FOXP2 antibodies revealed EdU+ cells were found in both LGE units and Region i/ii. Scale bar, 30 μm. (TIF) [file pbio.3001868.s011.tif]

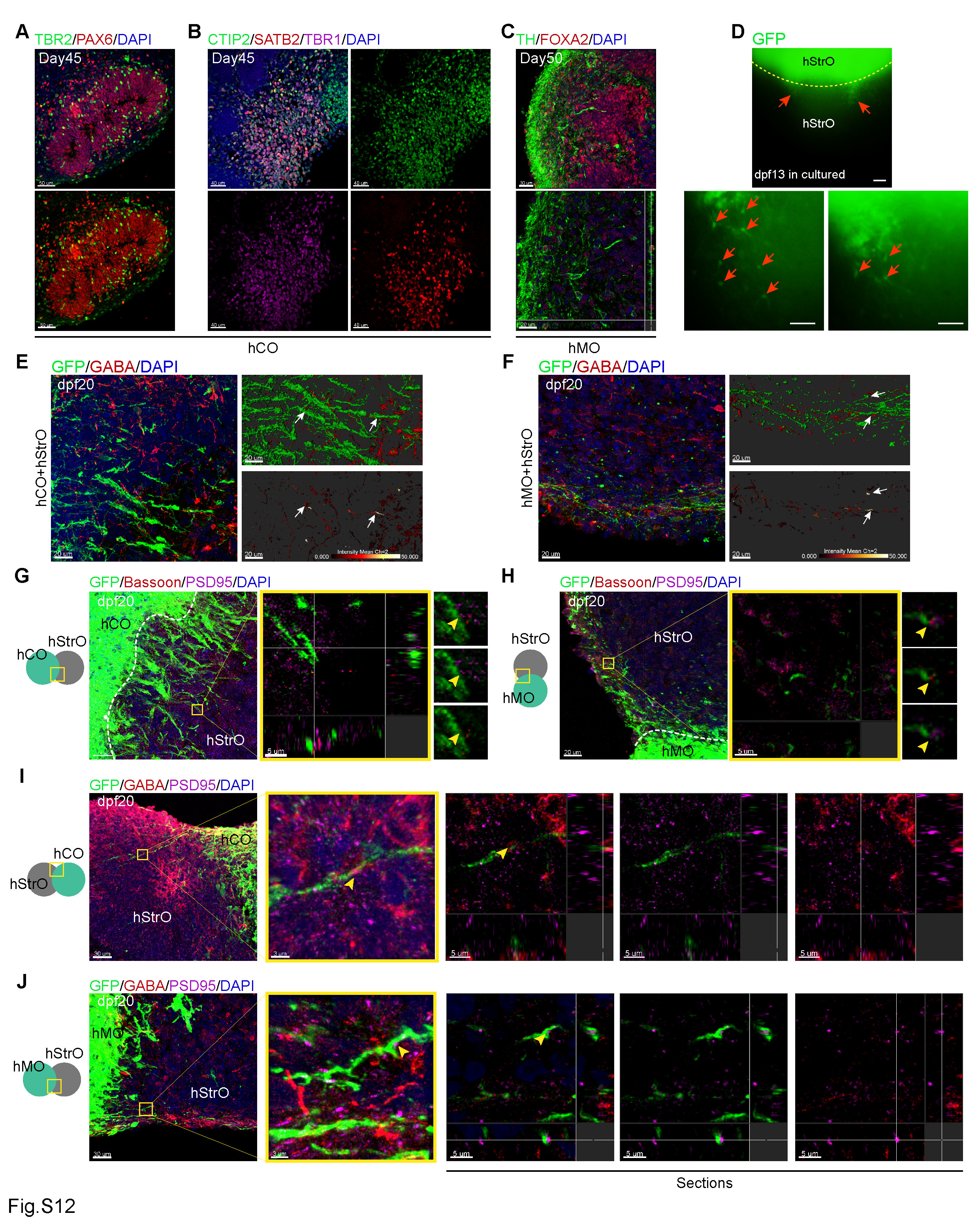

Supplement: S12 Fig — (A) Immunostaining for PAX6 and TBR2 antibodies on Day 45 hCOs. Scale bar, 50 μm. (B) Immunostaining for TBR1, CTIP2, and SATB2 antibodies in hCOs on Day 45. Scale bar, 40 μm. (C) Immunostaining images for FOXA2 and TH antibodies on Day 50 hMOs. The lower panel is the single ortho view of Z-stack images. Scale bar in the upper panel, 30 μm; in the lower panel, 30 μm. (D) Images of hStr-StrOs at dpf 13. The lower panels are the magnified images. Red arrows showed GFP+ migrating cells from hStrO. Scale bar, 100 μm. (E, F) Immunostaining for GFP and GABA antibodies revealed the intermingled GFP+ axons with GABA+ neurons in hC-StrOs and hM-StrOs at dpf 20. The left panels show the tomography of the boxed region rendered by Imaris. Scale bar, 20 μm. (G, H) Immunostaining for GFP, Bassoon, and PSD95 antibodies revealed contact between a pre- and postsynapse in hC-StrOs and hM-StrOs at dpf 20. Arrows showed the contact of Bassoon+ presynapses with PSD95+ postsynapses on GFP+ axons. Scale bar, G, 30 μm; H, 20 μm. (I, J) Immunostaining for GFP, GABA, and PSD95 antibodies revealed GFP+ axons projecting towards PSD95+ puncta on GABA neurons in hC-StrOs and hM-StrOs at dpf 20. Arrows showed the contact of GABA+ neurons with PSD95+ puncta in GFP+ axons. The right 3 panels show the relationship between the 2 signals. Scale bar, 30 μm. (TIF) [file pbio.3001868.s012.tif]

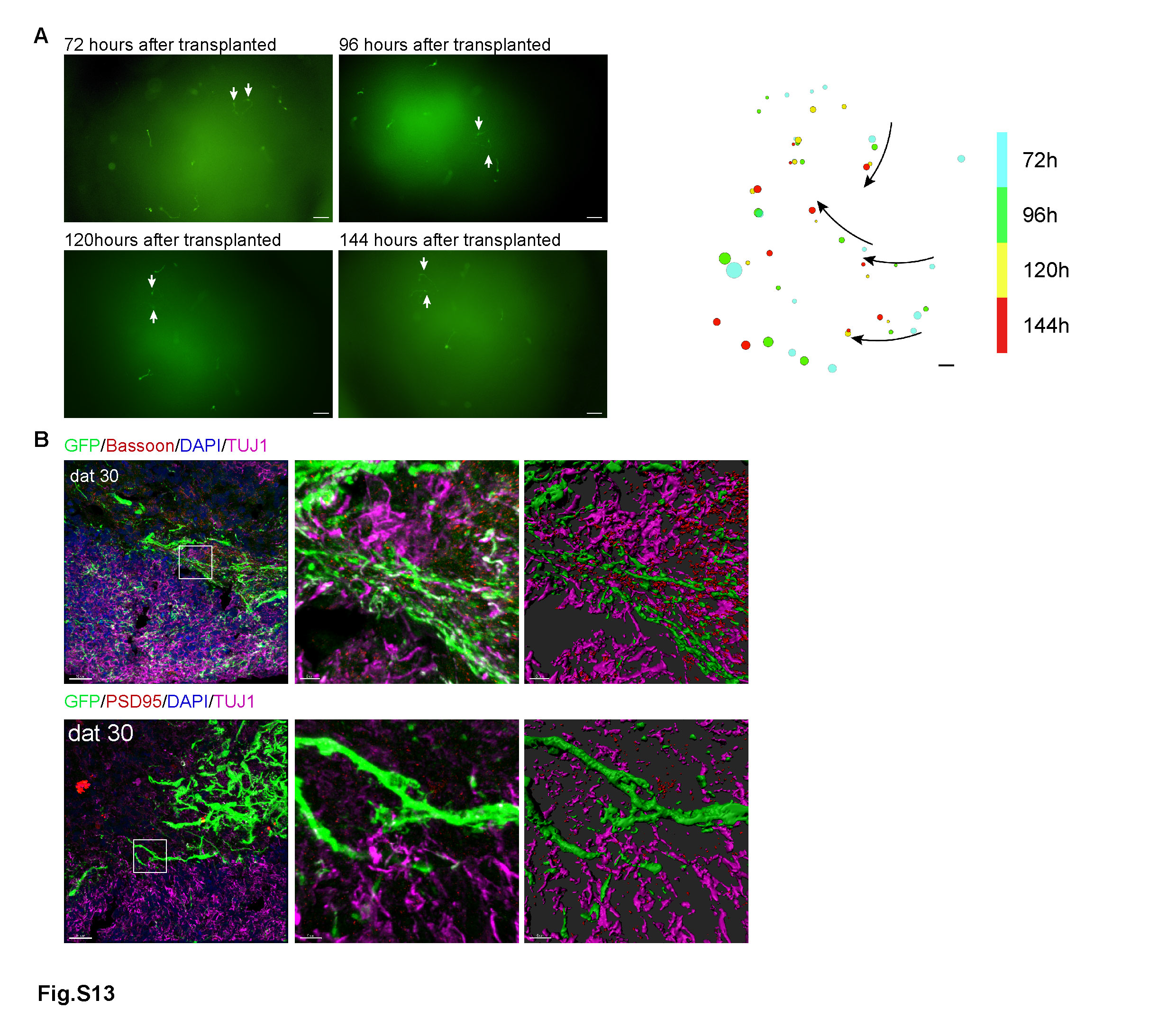

Supplement: S13 Fig — (A) Fluorescent image of hStrOs reveals that neurons exhibit migratory-like behavior after coculture. The right panel shows the migration trajectory plotted after overlapping the fluorescence photos at a different time (72, 96, 120, and 144 h). Scale bar, 200 μm. (B) Immunostaining for bassoon, PSD95, Tuj1, and GFP antibodies reveals the anatomical integration between GFP+ transplanted neurons and hStrO. The boxed region is the magnified region in the middle panel; the rendered tomography of fluorescent signals is in the right panel. dat, days after transplanted. Scale bar, 30 μm. (TIF) [file pbio.3001868.s013.tif]
