# Supplementary material for: Human striatal organoids derived from pluripotent stem cells recapitulate striatal development and compartments
Source: PLoS Biol. 2022 Nov 17;20(11):e3001868. doi: 10.1371/journal.pbio.3001868 (PMC9714809; doi:10.1371/journal.pbio.3001868)
Supplement: S1 Table — (PDF) [file pbio.3001868.s014.pdf]

Supplementary Table 1: Antibodies for immunostaining with their purpose.

Name	Purpose
General markers in human brain	
SOX2	Neural progenitor marker
Ki67	Proliferative cells marker
NEUN	Mature neurons marker
DCX	Immature neuron marker
TUJ1	Neuron marker
MAP2	Mature neurons marker
Bassoon	Pre-synaptic proteins marker
PSD95	Post-synaptic proteins marker
GFAP	Astrocytes marker
MBP	Myelin essential protein
PAX6	Neuroectoderm markers
SOX1	Neuroectoderm markers
OTX2	Forebrain marker
FOXG1	Forebrain marker
Region-specific markers in human brain	
Cortex	
PAX6	Dorsal forebrain progenitor marker
TBR1	One of cortical layer neuron markers
CTIP2	One of cortical layer neuron markers
SATB2	One of cortical layer neuron markers
LGE	
GSH2	LGE progenitor cells marker
MASH1	LGE Intermediate progenitor cells marker
DLX2	Ventral forebrain marker
GABA	GABAergic neuron marker
MEIS1/2	Striatal MSN marker
CTIP2	Striatal MSN marker
DARPP32	Striatal MSN marker/Striosome-enriched marker
FOXP2	Striosome-enriched marker
CALB2	Striosome-enriched marker
MOR1	Striosome-enriched marker
CALB1	Matrix-enriched marker
MGE	
NKX2.1	MGE marker
Mid-brain	
FOXA2	Midbrain marker
TH	Dopaminergic neurons marker
