# Supplementary material for: Human striatal organoids derived from pluripotent stem cells recapitulate striatal development and compartments
Source: PLoS Biol. 2022 Nov 17;20(11):e3001868. doi: 10.1371/journal.pbio.3001868 (PMC9714809; doi:10.1371/journal.pbio.3001868)
Supplement: S2 Table — (PDF) [file pbio.3001868.s015.pdf]

Supplementary Table 2:
Comparison of protocols for striatal neuron differentiation from human pluripotent stem cells

	Ma et al.,[1] 2012	Carri et al.,[2] 2013	Arber et al.,[3] 2015	hStrS Miura et al,[4] 2020	hStrO Chen et al
Type of culture	2D	2D	2D	3D	3D
Patterning	SHH or Purmorphamine	SHHC-25II DKK1	Activin A	Activin A IWP-2 SR11237	Purmorphamine
Cell diversity	Progenitors Neurons	Progenitors Neurons	Progenitors Neurons	Progenitors Neurons Astrocytes Oligodendrocytes	Progenitors Neurons Astrocytes Oligodendrocytes
Proportion of CTIP2+ cells	Not shown	60%	50%	50%	20%
Proportion of DARPP32+ neurons	80%	20% (of MAP2+ cells)	20-50%	30% (of NeuN+ cells)	70% (of NeuN+ cells)
Proportion of CTIP2+ DARPP32+ neurons	Not shown	Not shown	Not shown	Not shown	18.27±10.27%
Spatial cytoarchitecture	-	-	-	Not shown	VZ, SVZ, and MZ-like zones Compartment-like structure

Reference

1. Ma, L., et al., *Human embryonic stem cell-derived GABA neurons correct locomotion deficits in quinolinic acid-lesioned mice.* Cell Stem Cell, 2012. **10**(4): p. 455-464.
2. Delli Carri, A., et al., *Developmentally coordinated extrinsic signals drive human pluripotent stem cell differentiation toward authentic DARPP-32+ medium-sized spiny neurons.* Development (Cambridge, England), 2013. **140**(2): p. 301-312.
3. Arber, C., et al., *Activin A directs striatal projection neuron differentiation of human pluripotent stem cells.* Development (Cambridge, England), 2015. **142**(7): p. 1375-1386.
4. Miura, Y., et al., *Generation of human striatal organoids and cortico-striatal assembloids from human pluripotent stem cells.* Nature Biotechnology, 2020. **38**(12): p. 1421-1430.
