# Supplementary material for: Human striatal organoids derived from pluripotent stem cells recapitulate striatal development and compartments
Source: PLoS Biol. 2022 Nov 17;20(11):e3001868. doi: 10.1371/journal.pbio.3001868 (PMC9714809; doi:10.1371/journal.pbio.3001868)
Supplement: Attachment — Submitted filename: response+to+reviewer-PBIOLOGY-final-0808.docx [file pbio.3001868.s018.docx]

Dear editor and reviewers:

We greatly appreciate the three reviewers and editor for taking enormous time and effort to review our manuscript and providing a chance to improve our manuscript. We have revised this manuscript with full inspiration in the lockdown season based on reviewer suggestions and comments. We have addressed all concerns of three reviewers by point-to-point reply, which includes in response to reviewer letters and providing a document that included the characterization of HD cells as a reviewer-only figure. We hope that our additional efforts address all the concerns of reviewers and editor.

In the revised version, we provided a clean copy and highlighted copy. In highlighted copy, we have highlighted the changes in red.

Thanks again for reviewing and revising our manuscript. We hope that our efforts and revision fully address all concerns of the reviewer and Editor about our manuscript and meet the criteria for acceptance in PLOS Biology.

If you have any concerns about this manuscript, we are willing to address timely.

Sincerely

**Lixiang Ma,** MD, PhD. Associate Professor

Department of Anatomy and Histology & Embryology, School of Basic Medical Sciences, Fudan University, Shanghai 200032 China Email: lxma@fudan.edu.cn.

Rev. 1:

In this manuscript, Chen et al. used human pluripotent stem cells to generate human striatal organoids (hStrOs) that recapitulates the development of fetal striatum. Although hStrOs were generated and reported by other groups before, here authors comprehensively investigated the cellular composition and developmental time course of cellular and molecular changes through different stages of hStrO development with immunohistochemical and transcriptomics approaches. The method authors developed induced the development of distinct striatal compartments in a single organoid including striosomes and matrix. Also, using organoid fusions, they have identified neural migration/axon guidance differences between striatum and different brain regions including the cortex and the midbrain. Finally, they have transplanted Huntington's disease patient-derived striatal neurons into the day 45 hStrOs and showed successful engraftment of these neurons with healthy neurons that can be used as a model to investigate functional integration of donor neurons into the host.

Overall, the authors developed a new approach to generate striatum organoids with distinct striatal compartment, they had extensively characterized the cellular and molecular features, and they have shown that their hStrOs can be used to model cellular interactions with other brain regions, and these organoids can be used as a host for donor-host integration studies. Moreover, the manuscript is well written, and the figures are well-organized. There are only a couple of questions/suggestions which may improve the conclusions of the manuscript.

We greatly appreciate your suggestions. Based on your advice, we have revised our manuscript. In the revised version, we added the data of HD hStrO based on our systemic work in a big HD family and the current manuscript. Hopefully, we fully address the significance of HD cell transplantation in vitro, our revision fully addresses the concerns about this manuscript, and our data support the conclusion of this manuscript.

1. The striatum develops from the lateral ganglionic eminence (LGE), which is located at the ventral part of the developing forebrain. This region is known to express dorsal forebrain marker Pax6 at low levels, which is also supported by authors' data in Fig. 1D-F and Fig. S1B (<1% Pax6-positive cells). However, following these results, in Fig S2A-B, they showed that close to 90% of cells dissociated from hStrOs are Pax6-positive. Is this a discrepancy in their data? Is it due to the samples taken on different days in culture? Authors need to clarify this either in the result section or discuss it in the discussion section.

Response to reviewer #1-1

Thanks for your insightful comments. As we know, PAX6 was a classic neocortex-enriched differentially expressed gene (DEG). Another role of PAX6 is the earliest neuroectodermal marker expressed in the developing human central nervous system (CNS). It has been reported that, at 3 w + 4 d, PAX6 was expressed in the entire neuraxis^1^. At 7 W, PAX6 and GSX2 began to define the pallial-subpallial boundary (PSB) between neocortex and lateral ganglionic eminence^1^. In **Fig. S2A**, over 90% are PAX6+ cells dissociated from hStrOs after 20 days in culture, which represent the early stages of our differentiation protocol. Here, PAX6 is the neuroectodermal marker. While in **Fig. 1E, 1F and S1B**, our results showed the Pax6 at low levels in day 45 hStrOs. Here, PAX6 defined the dorsal forebrain fate. Moreover, day 45 hStrOs also showed the high-level expression of GSH2 (GSX2) **(Fig. 2C)**, indicating the LGE fates. Hopefully, this explanation addresses your concern here.

2. In the introduction section, line 8, authors used the terms 'self-patterning' and 'unself-patterning' possibly referring to the generation of region-specific vs cerebral organoids through guided vs unguided patterning protocols. Please correct these terms as "guided and unguided methods" since unself-patterning is not really a term to describe unguided methods.

Response to reviewer #1-2

Thanks for pointing this out. We have corrected these terms in the revised version.

3. In general, authors never discussed in their manuscript the strength of their method to generate hStrOs over the previous methods. It would be nice to add a couple of sentence regarding this at least in the Discussion.

Response to reviewer #1-3

We included a supplementary table in our results **(Supplementary Table 2)**, including the comparison with previous approaches for deriving striatal neurons both in 2D and 3D in the revised version. We fully discussed the strength in our methods in the revised version, **please refer to our discussion, page 19**.

4. As they mentioned in the Discussion, rather than only showing pre- and post-synaptic marker staining data in fused organoids, an electrophysiological approach or calcium imaging would better show the maturation of striatal neurons with or without input from cortical/midbrain neurons.

Response to reviewer #1-4

Thanks for pointing this out. We agree that the electrophysiological approach or calcium imaging would better show the maturation of striatal neurons in hStrOs and strengthen the conclusion of our work. As you know, setting up an electrophysiological approach or calcium imaging platform in hStrOs needs enormous efforts and a normal environment without frequent lockdown (the accesses to these facilities are completely locked during the lockdown), we are beginning to set up a calcium imaging platform recently, and we will systemically and physiologically characterize hStrO in the future. We believe that you will see these data soon but not in this paper. We wish our revision fully or sufficiently supports the conclusion in this work.

5. Although the injection of patient-derived neurons and subsequent migratory characteristics of them in hStrOs show that hStrOs can serve as a host for these neurons, I do not totally grasp the importance of this data and how it makes their hStrOs a better model. This is a process which can possibly occur in any type of organoids. Please discuss the importance of this experiment and what it proves.

Response to reviewer #1-5

This question is also raised by Reviewer #3. The only aim of graft HD cells is to expand the utility of hStrOs. Thus we selected HD patient cells with GFP for evolving iPS cells derived neurons in the host. As the transplantation window of the iPS-derived neuron is difficult to evaluate, it will be helpful for our next stage and others who apply stem cell transplantation in the brain, if possible. We have constructed iPSs of 10 HD patients from a big family in China. You will see the systemic study of these cell lines and the progress of this research near future. **(Please refer to reviewer-only images 1-2 and table)**. Despite our previous approach of transplanting ES-derived neurons is promising^2^, we urgently wish to see the evolution of transplanted cells in the host. We hope this tool provides us with some information to improve our approach.

In addition, replacing irreversible effete neurons is the only way to cure or mitigate multiple neurodegenerative disorders, including HD, via transplanting iPS or ES-derived neurons. Systemic evaluation of the candidate cells is a necessary step before we move to the therapeutic approach. You might agree with our view that systemic evaluation needs more tangible tools. hStrOs at least provide a tool to visualize the behavior of candidate cells, including survival, death, migration, and maturation. Notably, HD graft also adheres to the self-organizing regionalization in hStrOs. It implies the potential utility of our hStrOs **(Please refer to our discussion,** **page 21)**. Your hypothesis might be correct that the phenome which we observed might be common in any type of organoid. However, the maintaining system of brain organoids contains the supporting components of specific neurons, and we thought that hStrOs are better than other brain organoids for testing the biological behavior of MSNs, which are transplanted to the brain. In the future, the updated hStrOs with microglia and circulation might provide us with a good tool to evaluate the biological behavior of transplanted candidate cells. We agree that our organoids are preliminary. However, it has the potential to update and promise. **(Please refer to reviewer-only images 1-2 and table)**

Rev. 2:

In this manuscript, the authors are characterizing human iPSC and stem cell derived organoids (in isolation and fused, relative to region) to identify conditions for improved development of striatal organoids which better represent the cell types and physical compartmentalization of the LGE-derived striatum. The main variable is concentration of puromorphine, which they confirm is required for "striatal" development from iPSCs. This reviewer found it extremely difficult to read and follow this manuscript due to jumping around between markers, supposed regions of the organoids, overuse of supplementary data for key points (e.g. regionalization) and the number of small and difficult to interpret figures some of which are not labeled with what they are stained. Also, this reviewer is very sympathetic to the fact that English is not everybody's first language, but the grammar errors herein actually sometimes make the text difficult to understand, and it would have been helpful to have the language edited prior to review. The manuscript would also really benefit by an initial Table 1 listing the markers that are used and what they are purported to represent. That being said, the main conclusions are that the sonic hedgehog pathway is required for regionalization, i.e. that puromorphine was required, and that compartment and regional specific synapses are visualized in fused organoids.

Response to reviewer #2-1

Thanks for pointing these out. We reorganized our figures to make them readable in the revised version. We renamed the supposed regions of the organoids in the revised version. It looks simple now. We included a table that described the markers which are used in the revised version **(please refer to Supplementary Table 1)**. Before resubmitting the manuscript, we used the Elsevier language editing service to improve our manuscript in the revised version. Hopefully, our efforts address the concerns of the reviewer here.

There is a theoretical problem in claiming compartmentalization, which appears to be primarily based on visual distinctions between what is first called "hyaline patches" not seen in cortical organoids, and then by regionalization noted by early and late LGE markers and two regions which differed in cell density. There is a problem here in that DARPP-32 is used as the definitive marker of MSNs in all regions, so it is not clear what is really distinguishing the regions neurochemically. Single cell RNA seq is not adequate for this purpose as the clusters are not spatially defined. More problematic, and apologies if this was missed, all DARPP-32+ cells should be GABAergic if they are to be defined as MSNs, and several figures, particularly in Figure 2, do not appear to show that this is the case. Of course it goes without saying also that all DARPP-32+ and CTIP2 (better referred to as Bcl11b) should be NeuN+, and the lack of double label with DARPP32 and MAP2 is also confusing.

Response to reviewer #2-2

As you know, the definition of putative compartmentalization in hStrO is from the visual distinctions, including "hyaline patches" and different cell densities, which we term Self-organized regionalization. You are right that single-cell RNA seq is inadequate to identify a cluster's spatial information in hStrO. In the revised version, we turn to intensive immunostainings and rigorous counting to provide robust evidence in neurochemical and developmental properties for specific regions that we defined. For example, we introduced more striatal compartmental markers in immunostainings, including CALB1, CALB2, and MOR1 based on the reviewer's suggestion **(please refer to Fig. 3F and 4A)**; we investigated the progenitors or post-mitotic neuronal diversity in regionalized hStrOs **(please refer to Fig. 3J, 3K and S8)**. Our results suggested an underlying mechanism of hStrO regionalization partially recapitulating striatal compartmental and MSN-specific rules. Hopefully, our efforts and data in the revised version fully/significantly addressed your concerns.

As we know, the ratio of MSNs is a criterion for evaluating the striatal derivation protocol. Our focuses are to identify hStrOs neuronal types in our protocol. Co-immunostaining with DARPP32 and MAP2 showed in both **Fig. 1 and Fig. 2** of the previous submission. To address your concerns, we updated the identification of hStrOs neuron types in the revised version: In the revised version, typical MASH1+/CTIP2+/GABA+ neurons on day 45 hStrOs were shown **(Fig. 2E)**. Moreover, we showed typical DARPP32+ neurons co-expressed GABA or CTIP2 in day100 hStrOs and the mature MSN% in hStrOs (CTIP2+DARPP32+/NEUN+%; 18.27±10.27%, day 100) **(Fig. 2F)**. We also provide a table **(Supplementary Table 2)** to compare different protocol.

Part of the problem lies in the fact that DARPP-32 is indeed initially a marker of striosome cells in vivo, but then its expression becomes generalized, and although the authors refer to the fact that striosome markers change with development, they make no effort to explain to the reader what phase of development they think they are modeling or characterizing. Within the striosome regions, the authors then should demonstrate double label of DARPP-32/FoxP2. Also if there are specific striosome regions, then presumably the authors are thinking that the remaining striatal-like portion of the organoid represents matrix? but again this is characterized only by scRNA seq. It should also be noted that in early striatal development, calbindin localizes more to striosomes than matrix, again making interpretation here difficult vis a vis what developmental period the authors think each time point represents.

Response to reviewer #2-3

Thanks for pointing this out. We appreciate your critical reading and totally agree the points which you mentioned. You are correct that DARPP-32 defined the early striosome in vivo, so does FOXP2. The compartmentalized region on day 45 is a preliminary structure. We have rigorously stained, characterized, and annotated these regions in the revised version **(please refer to Fig. 4)**. In vivo, the compartmentalized region with mosaic appearance is originated from regulated lineage progression neural progenitors, the mosaic patterns involved with migration, integration, even the cross-talk between other parts of the brain^3,4^. Based on this fact, hStrO is a preliminary model that lacks a precise modification by multiple factors in the brain.

Thus, we considered Region ii as the matrix-like zones. We do observe CALB1 enriched in Region ii. During fetal brain development, CALB1 is periodically unregulated in the striosome, which might be related to integrating signals from the different brain regions^5-12^. We included CALB1 staining data in the revised version. Hopefully, these data fully or at least sufficiently address your concerns here.

It is challenging to comment specifically on the experiments with "HD cells" due to the lack of characterization of these cells. Likewise, the specificity of connections in the fused organoids is difficult to validate without the same characterization re dopaminergic neurons, etc, as was performed in isolated organoids.

Response to reviewer #2-4

This question is also raised by Reviewer #3. The only aim of graft HD cells is to expand the utility of hStrOs. Thus we selected HD patient cells with GFP for evolving iPS cells derived neurons in the host. As the transplantation window of the iPS-derived neuron is difficult to evaluate, it will be helpful for our next stage and others who apply stem cell transplantation in the brain. We have constructed iPS of 10 HD patients from a big family in China. You will see the systemic study of these cell lines and the progresses of this research near future. Reviewer 1 has the same view on this part as us. The characterization of HD-hStrO is updated in **Fig. 6**. The systemic characterization of the HD cells and family HD cells will appear in another work (under review). **(Please refer to reviewer-only images 1-2 and table)**

We constructed hMOs by using the published methods^13^. Figure S12C showed the characterization of dopaminergic neurons. As we know, FOXA2 is a marker of floor plate progenitors in the midbrain, which also express mature dopaminergic neurons.

Rev. 3:

The manuscript by Chen et al titled "Human striatal organoids derived from pluripotent stem cells recapitulate striatal development and compartments" describes a method to generate striatal organoids containing DARPP32 cells and regions that appear to resemble the striatum striosome and matrix compartments. In addition, the authors describe striatal assembloids that show projections that appear to be similar to projection patterns in vivo. Because striatal organoids and assembloids have already been described by a different group (Miura et al 2020), the main novelty of the work is the formation of discrete striosome and matrix compartments. In vivo these compartments are characterized by distinct histological markers and afferent and efferent connectivity. In the manuscript, however, the characterization of these compartments in organoids was not thorough or convincing. More specific concerns are described below:

Response to reviewer #3

We further characterized the self-organizing regionalization in hStrO. Updated evidences include more compartmental markers and specific developmental processes.

1) While this reviewer appreciates the authors' effort to quantify a big number of striatal markers by immunohistochemistry, there are some issues with the quantification:

- The numbers in Figures 1 and 2 do not seem to match. For example, Figure 1 shows that ~70% of DAPI at d45 are DARPP32+, yet Figure 2 and S2 show that ~60% of DAPI at this age are SOX2. I would not expect DARPP32 cells to be SOX2+. In another example, Figure 2 shows that ~80% of DARPP32 cells are NeuN+, yet NeuN cells only account for 5% of all DAPI. Are most DARPP32 cells NeuN negative then?

Response to reviewer #3-1

Thanks for pointing out these questions about counting. Our counting method and figure seem to bring confusion to you. Thus, we have updated this part in the revised version. We simplified the statistical graphs and removed some of the non-important graphs in the revised version. Hopefully, our update fully addresses the concern.

**The adjustments in the revised version:**

**Previous version: Fig. 1H**. DARPP32+ cells are the neurons which locate outside of rosettes. We have only counted DARPP32+ cells outside the rosettes.

**In the revised version: Fig. 1I**; We only used the proportion of DARPP32 in MAP2-positive cells to represent MSN neurons.

**Previous version:** **Fig. 2H**. DARPP32+NEUN+/NEUN+% =~80%

**In the revised version:** **Fig. 2E**. We highlight this in the figure legend. The count means the proportion of DARPP32+ NEUN+ double-positive cells in NEUN-positive cells

**Previous version:** **Fig. 2M-R**

**In the revised version:** Removed. We do not consider this part of the statistics to be helpful for the manuscript.

**Previous version:** **Fig. 3J and 3L**

**In the revised version:** Removed. We do not consider this part of the statistics to be helpful for the manuscript. We do not consider **Fig. 3J** to be helpful for the manuscript. And **Fig. 3L** duplicates **Fig. 3M (old version)/Fig. S7D (new version)**

- It is unclear what the points in each graph represent. Figure legends say "n = 4-6 organoids", yet there are a lot more points in each graph. If various cryosections are being quantified per organoid, then these need to be averaged and each point should represent an organoid and statistics should be performed with organoids as n.

Thanks for pointing this out. You are right. Each group contained 4-6 organoids, and 4-5 sections of each organoid were analyzed. Thus, many more points in each group were seen in each graph or bar. Based on your suggestions, we have adjusted these graphs in the revised version.

2) The authors make a claim about the position of cells within an organoid; however, this should be quantified. For example, although one image shows MASH1 cells in "SVZ", and CTIP2 cells in "MZ", Figure 2F shows CTIP2 cells colocalizing with MASH1 in the SVZ.

Response to reviewer #3-2

Thanks for pointing this out. We quantified the distribution of striatal markers in each supposed layer and included that in the revised version **(Fig. 2C and 2D)**.

3) Parts of figure 1 and 2 seem to be repeating themselves, they can be consolidated and reorganized (currently the flow is not very clear and jumps from progenitors to neurons and back for no clear reason). Either have the single cell as a separate figure, or have a figure for progenitors and another for neurons

Response to reviewer #3-3

We have updated the alignment of **Figure 1** with **Figure 2,** making it easy to read. **Figure 1** focuses on the role of Pur in 3D cultures, and **Figure 2** provides a more detailed characterization of striatal fate in hStrOs**.**

4) The authors should explain the rationale of doing most of the characterization of the organoids at d45 yet performing scRNAseq at d110.

Response to reviewer #3-4

Thanks for raising this important question. Actually, we have not only characterized day 45 organoids but in other time points, including days 60, 80, and 100 in the revised version. The reason for performing scRNAseq at day 110 is that we previously found that the MSNs mature around 100 days in 2D culture and show typical morphological and physiological signatures of mature MSNs in vivo^2^.

5) Quantification data for MASH1 at d45 in Figures 2E and 2N looks very similar. Please double-check that the same data was not used for both graphs.

Response to reviewer #3-5

Thanks for pointing this out. It should be identical. MASH1 counting included the data of multiple times, and we used the data of multiple to compare. Thus, we displayed that several times. In the revised version, we removed these repeats.

6) The rationale behind the characterization of striosome and matrix compartments was not clear, and future markers and quantification would be needed to claim these two compartments are formed in organoids. For example:

Response to reviewer #3-6

- In figure 3B - why are KI67 negative areas called mature regions? They are not co-stained with any other marker that would suggest they are "mature"

Thanks for pointing this out. We agree that the Ki67 negative areas (Region i/ii) cannot be called the mature regions due to the scattered SOX2+ cells **(Fig. 4B)**. Co-labeling of Ki67 with MAP2 revealed the post-mitotic identity of these regions. In the revised version, we have updated the terms of self-organized regionalization in hStrOs **(Fig. 3)**.

- Moreover, it looks like FOXP2 cells in "mature region I" are MAP2 negative (Fig 3H, I). Is that expected?

Our results supported those different progenitors with multiple origins involved with self-regionalization in hStrOs, resembling LGE formation in vivo. These kinds of regionalization occur after mitosis of neuronal progenitors, and the post-mitotic neurons need a period to mature MAP+ fibers in Region i and Region ii that increase via a culturing time-dependent way **(Fig. 4B and 4C)**. We also noticed that DCX+GABA+ cells in hStrOs which are negative in MAP2 staining, displayed a slender morphology that is a characteristic of migrating neurons **(Fig. 4E and 4F)** some newly formed neurons in hStrO with the slender body were not labeled by MAP2 **(Fig. 4G)**. Thus, the results are expected.

- In Figure 3D - Why are DARPP32 regions MAP2 negative? Based on previous figures most DARPP32 cells should be MAP2 positive

Thanks for pointing this out. DARPP32+ is expressed in both immature and mature GABAergic neurons, whereas MAP2 is only expressed in mature neurons. Our quantification showed the expression of DARPP32 in mature neurons and does not indicate that most DARPP32 cells are mature neurons.

- In the scRNAseq dataset, while there is expression of striosome/matrix genes, they are scattered and they don't seem to be colocalized. Eg. ID4 and CALB1 are not expressed in the same cells and OPRM1 and SEMA5B are not expressed in same cells

As reviewer #2 mentioned, single-cell RNA seq cannot accurately provide information about the spatial pattern of clusters. Thus, we removed these data from our manuscript. In the revised version, we did intensive immunostaining to provide more precise information about specific regions' neurochemical and developmental properties.

- How many organoids was the regionalization seen in? (Fig 3F,G) This is not specified in the legend.

To investigate the prevalence of self-organized regionalization in hStrO, we sampled a random batch of hStrO in cultured (Day 40), and the results showed that 31 of total 35 organoids showed significant hyaline patch **(Fig. S6A and S6B)**. We also sampled frozen sections collected from random batches of Day45 hStrOs and found that 19 of 24 different organoids showed a clear FOXP2 enriched Region i **(Fig. S6C and S6D)**.

- Striosome and matrix compartments are characterized by the expression of a number of markers. Have the authors checked the expression of DRD1 and DRD2, GAD1, SST, TAC1, PENK, etc? It would be important to show more markers for these regions

We detected additional compartmental markers, including CALB1, CALB2, and MOR1^14^, in the revised version **(Fig. 3F, 4A, S9A and S9B)**.

7) Although it is in principle an interesting idea to graft HD cells, it is unclear what the purpose of this experiment was. Why not use control GFP+ cells?

Response to reviewer #3-7

The question is also raised by reviewer #2. The only aim of graft HD cells is to expand the utility of hStrOs. Thus we selected HD patient cells with GFP for evolving iPS cells derived neurons in the host. As the transplantation window of iPS-derived neurons is difficult to evaluate, it will be helpful for our next stage. We have constructed near iPS of 10 HD patients from a big family in China. You will see the systemic study of these cell lines and the progress of this research near future. Reviewer 1 has the same view on this part as us.

Minor

- In introduction: unself-patterning - there is not such a thing as unself-patterning

Response:

This question is also raised by reviewer #1. The reviewer #1 advised to use "guided and unguided methods". Based on reviewer # suggestion, we have replaced 'self-patterning' and 'unself-patterning' with "guided and unguided methods" in the revised version.

- In introduction: "spatially self-organized and structurally and functionally recapitulate the neuronal activities of the human brain" -> tone down

Response:

The sentence is a little overstated. We have tone down this sentence in the revised version.

- DARPP32 staining organoids in Figure 1G is not convincing - better picture would be ideal. Figure S2E has a better staining

Response:

Thanks for pointing this out. We have provided a clear image with stitching and inserts in the revised version **(please refer to Fig. 1H in the revised version)**.

- DLX2 is also an MGE marker yet it is being used as an exclusive LGE marker

Response:

You are right. Actually, except for NKX2-1, DLX2 is also expressed in MGE. We removed this result.

- Looking at the figures and figure legends, it is unclear when 2D vs 3D is being shown/quantified. Please specify clearly

Response:

The cell dissociated from hStrOs is 2D, and the rest of the results are based on 3D culture. We have highlighted the cell dissociated from hStrOs in figure **(Fig. 1C and S2A)**. We have specified the 2D and 3D counting in images.

- SOX2 staining looks unspecific. Note that it appears to be expressed in VZ and outside VZ (where MAP2 neurons are). I would not expect MAP2 neurons to be SOX2+

Response:

Thanks for pointing this out here. SOX2 is a transcription factor located in the nucleus. The reason for SOX2 appearing to be expressed in VZ and outside VZ might be that the partially tangling of MAP2 positive projections with SOX2+ progenitors cause the impression that SOX2 and MAP2 are together in lower magnification. However, SOX2 and MAP2 signals in organoids are completely spliced in high-resolution images with Z-stack **(please refer to Fig. S10A)**. The counting of SOX2 and NEUN positive cells further showed the specificity of SOX2 **(Fig. S2B and S2C)**. SVZ is the largest in LGE. Thus it is expected to mixing of SOX2+ cells with MAP2+ projections.

- Clarify what Fig 2E is showing. The text implies that it is quantifying the position of GSX2/MASH1 and CTIP2 cells within the organoid, yet the figure legend indicates it is showing quantification of expression. "We found that cells labeled by different markers have their own preferences in the location: GSH2+ cells, mostly located in VZ-like zones (33.03 ± 11.84%); MASH1+ cells, sprinkled closed to the VZ-like zones (23.8 ± 9.977%); CTIP2+ cells, largely excluded from VZ-like zones (42.62 ± 12.83%)"

Response:

Thanks for pointing this out. We further quantified the distribution of striatal markers in each layer **(Fig. 2C and 2D)**.

- Gene names in single cell analysis should be italic

Response:

We have rewritten gene names in italic in the revised version.

- Specify what the colors represent in Fig 3L

Response:

We have removed **Fig. 3L(old version)** in the revised version.

- Images in 4B do not clearly depict projections and or migrating neurons. Except for one of the arrows where one can see a projection, it is hard to see what the rest of the arrows are pointing at

Response: Thanks for raising these questions. We have adjusted and updated these images in the revised version.

- Significance is not shown in Fig 4I

Response:

Thanks. We have added the significance in **Fig. 4I (old version)** in the revised version **(Fig. 5l)**.

- "it is rational to consider these results as reminiscent of the striosomal MSNs receiving preferential inputs from a deep cortical layer in vivo" - authors do not show projections being from deep or superficial cortical neurons.

Response:

Thanks again. We have rewritten the sentence in the revised version.

**Reference**

1.	Onorati M, Castiglioni V, Biasci D, et al. Molecular and functional definition of the developing human striatum. *Nat Neurosci* 2014; **17**(12): 1804-15.

2.	Ma L, Hu B, Liu Y, et al. Human embryonic stem cell-derived GABA neurons correct locomotion deficits in quinolinic acid-lesioned mice. *Cell Stem Cell* 2012; **10**(4): 455-64.

3.	Lebouc M, Richard Q, Garret M, Baufreton J. Striatal circuit development and its alterations in Huntington's disease. *Neurobiol Dis* 2020; **145**: 105076.

4.	Tinterri A, Menardy F, Diana MA, et al. Active intermixing of indirect and direct neurons builds the striatal mosaic. *Nat Commun* 2018; **9**(1): 4725.

5.	Holt DJ, Graybiel AM, Saper CB. Neurochemical architecture of the human striatum. *J Comp Neurol* 1997; **384**(1): 1-25.

6.	Letinić K, Kostović I. Transient patterns of calbindin-D28k expression in the developing striatum of man. *Neurosci Lett* 1996; **220**(3): 211-4.

7.	Prensa L, Giménez-Amaya JM, Parent A. Chemical heterogeneity of the striosomal compartment in the human striatum. *J Comp Neurol* 1999; **413**(4): 603-18.

8.	Liu FC, Graybiel AM. Heterogeneous development of calbindin-D28K expression in the striatal matrix. *J Comp Neurol* 1992; **320**(3): 304-22.

9.	Kubota Y, Kawaguchi Y. Spatial distributions of chemically identified intrinsic neurons in relation to patch and matrix compartments of rat neostriatum. *J Comp Neurol* 1993; **332**(4): 499-513.

10.	Olson L, Seiger A, Fuxe K. Heterogeneity of striatal and limbic dopamine innervation: highly fluorescent islands in developing and adult rats. *Brain Res* 1972; **44**(1): 283-8.

11.	Graybiel AM. Correspondence between the dopamine islands and striosomes of the mammalian striatum. *Neuroscience* 1984; **13**(4): 1157-87.

12.	Nishikawa S, Goto S, Yamada K, Hamasaki T, Ushio Y. Lack of Reelin causes malpositioning of nigral dopaminergic neurons: evidence from comparison of normal and Reln(rl) mutant mice. *J Comp Neurol* 2003; **461**(2): 166-73.

13.	Qian X, Jacob F, Song MM, Nguyen HN, Song H, Ming GL. Generation of human brain region-specific organoids using a miniaturized spinning bioreactor. *Nat Protoc* 2018; **13**(3): 565-80.

14.	Crittenden JR, Graybiel AM. Basal Ganglia disorders associated with imbalances in the striatal striosome and matrix compartments. *Front Neuroanat* 2011; **5**: 59.
